# Bortezomib promotes KHSV and EBV lytic cycle by activating JNK and autophagy

**DOI:** 10.1038/s41598-017-13533-7

**Published:** 2017-10-12

**Authors:** Marisa Granato, Maria Anele Romeo, Mariangela Sara Tiano, Roberta Santarelli, Roberta Gonnella, Maria Saveria Gilardini Montani, Alberto Faggioni, Mara Cirone

**Affiliations:** Department of Experimental Medicine, “Sapienza” University of Rome, Viale Regina Elena 324, 00161 Rome, Italy

## Abstract

KSHV and EBV are gammaherpesviruses strictly linked to human cancers. Even if the majority of cancer cells harbor a latent infection, the few cells that undergo viral replication may contribute to the pathogenesis and maintenance of the virus-associated malignancies. Cytotoxic drugs used for the therapies of cancers harboring virus-infection often have, as side effect, the activation of viral lytic cycle. Therefore it is important to investigate whether they affect viral reactivation and understand the underlying mechanisms involved. In this study, we found that proteasome inhibitor bortezomib, a cytotoxic drug that efficiently target gammaherpesvirus-associated B cell lymphomas, triggered KSHV or EBV viral lytic cycle by activating JNK, in the course of ER stress, and inducing autophagy. These results suggest that the manipulation of these pathways could limit viral spread and improve the outcome of bortezomib treatment in patients affected by gammaherpesvirus-associated lymphomas.

## Introduction

Tumor cells arising from Epstein-Barr virus (EBV) and Kaposi’s sarcoma-associated herpesvirus (KSHV)-associated cancers usually harbor latent infection and the few cells that undergo spontaneous viral replication promote viral spread and disease maintenance^1,2^. Viral reactivation can be induced upon appropriate stimuli that are usually cytotoxic^3^, although herpesvirus lytic proteins may partially counteract their cytotoxic effects. ER stress is one of the stimuli able to induce the reactivation from latency of KSHV^4,5^ and EBV^6^, two gammaherpesviruses strictly associated to Primary Effusion Lymphoma (PEL) and endemic Burkitt’s lymphoma (eBL), respectively. Endoplasmic Reticulum (ER) stress, triggered by perturbations within the ER that alter protein folding and processing, leads to the activation of the unfolded protein response (UPR)^7^. It is initiated by three ER transmembrane receptors: protein kinase RNA (PKR)-like ER kinase (PERK), inositol-requiring enzyme 1 (IRE1α) and activating transcription factor 6 (ATF6) that promote or cell survival or cell death depending on the balance between Binding immunoglobulin Protein/(78 kDa glucose regulated protein) (BiP/(GRP78) and C/EBP-Homologous Protein^8^. The underlying mechanisms of the ER stress-mediated gammaherpesvirus reactivation from latency are not fully clarified yet. Activating factor 4 (ATF4) and X-box binding protein 1 (XBP1) have been shown to be involved in KSHV replication induced by several ER stressors^5^. Conversely, in another study, IRE1α and PERK, ER stress sensors, is shown to be down regulated in PEL cells during the latent phase. CCAAT/enhancer-binding protein β (C/EBPβ) molecule, also up-regulated by ER stress^9^, has been shown to be involved in bortezomib-induced EBV lytic cycle activation^10^. ER stress is indeed induced by bortezomib, a peptide boronate, which inhibits 26 S proteasome leading to the accumulation of misfolded proteins not longer degraded via proteasome^11^. In the course of ER stress induced by bortezomib, c-Jun N-terminal Protein Kinase (JNK) can be activated by the IRE1 arm of UPR and may promote autophagy by phosphorylating Bcl-2^12^. Accordingly, we have previously shown that bortezomib-induced ER stress promoted a pro-survival autophagy through the activation of JNK in PEL cells^13^. Autophagy is a catabolic process that usually helps cancer cells to survive in stressful conditions such as during starvation or in the course of chemotherapies. Autophagy may also promote the lytic cycle of different viruses including gammaherpesviruses^14–17^. In this study, we investigated whether JNK activation by bortezomib could promote viral lytic cycle through autophagy induction in B cells latently infected with EBV or KSHV. JNK and the other Mitogen-Activated Protein Kinases (MAPKs) activated by cellular stress^18^ have been reported, in separate studies, to regulate the autophagic pathway^19^ and promote the viral lytic cycle^20,21^. However, the link between MAPKs and autophagy in the activation of gammaherpesvirus replication by bortezomib has never been explored. Understanding the molecular mechanisms underlying chemotherapy-induced viral reactivation from latency could help to achieve a better control of EBV and KSHV-associated malignancies, since their pathogenesis is strictly dependent on both latent and lytic phases of the viral life^22–24^.

## Results

### Bortezomib induces KSHV and EBV lytic antigen expression concomitantly with autophagy induction in cells undergoing apoptosis

**Figure 1:**
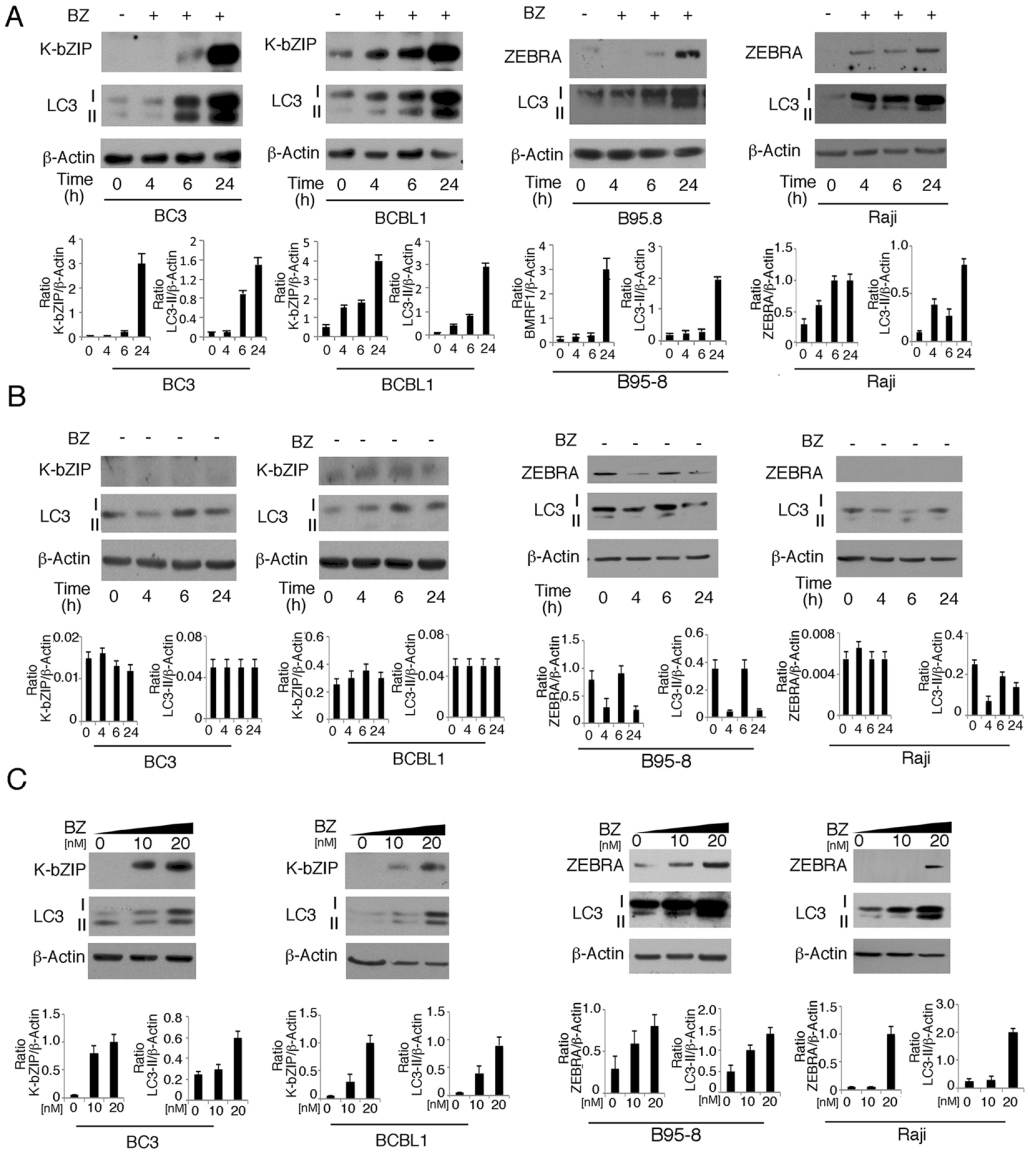
Bortezomib triggers KSHV and EBV lytic cycle concomitantly with autophagy induction in viral-infected B cells. (**A**) BC3, BCBL1, B95-8 and Raji cells were treated with bortezomib (BZ) (20 nM) for the indicated times and K-bZIP KSHV or ZEBRA EBV lytic antigen expression was evaluated by western blot analysis. (**B**) BC3, BCBL1, B95-8 and Raji cells were mock treated for the indicated times and K-bZIP or ZEBRA expression was evaluated by western blot analysis. (**C**) BC3, BCBL1, B95-8 and Raji cells were treated with different bortezomib doses (0-10-20 nM) for 24 hours and K-bZIP or ZEBRA lytic antigen expression was evaluated by western blot analysis. β-Actin was used as loading control. The histograms represent the mean plus standard deviation (SD) of the densitometric analysis of the ratio of specific proteins on β-Actin of three different experiments.

**Figure 2:**
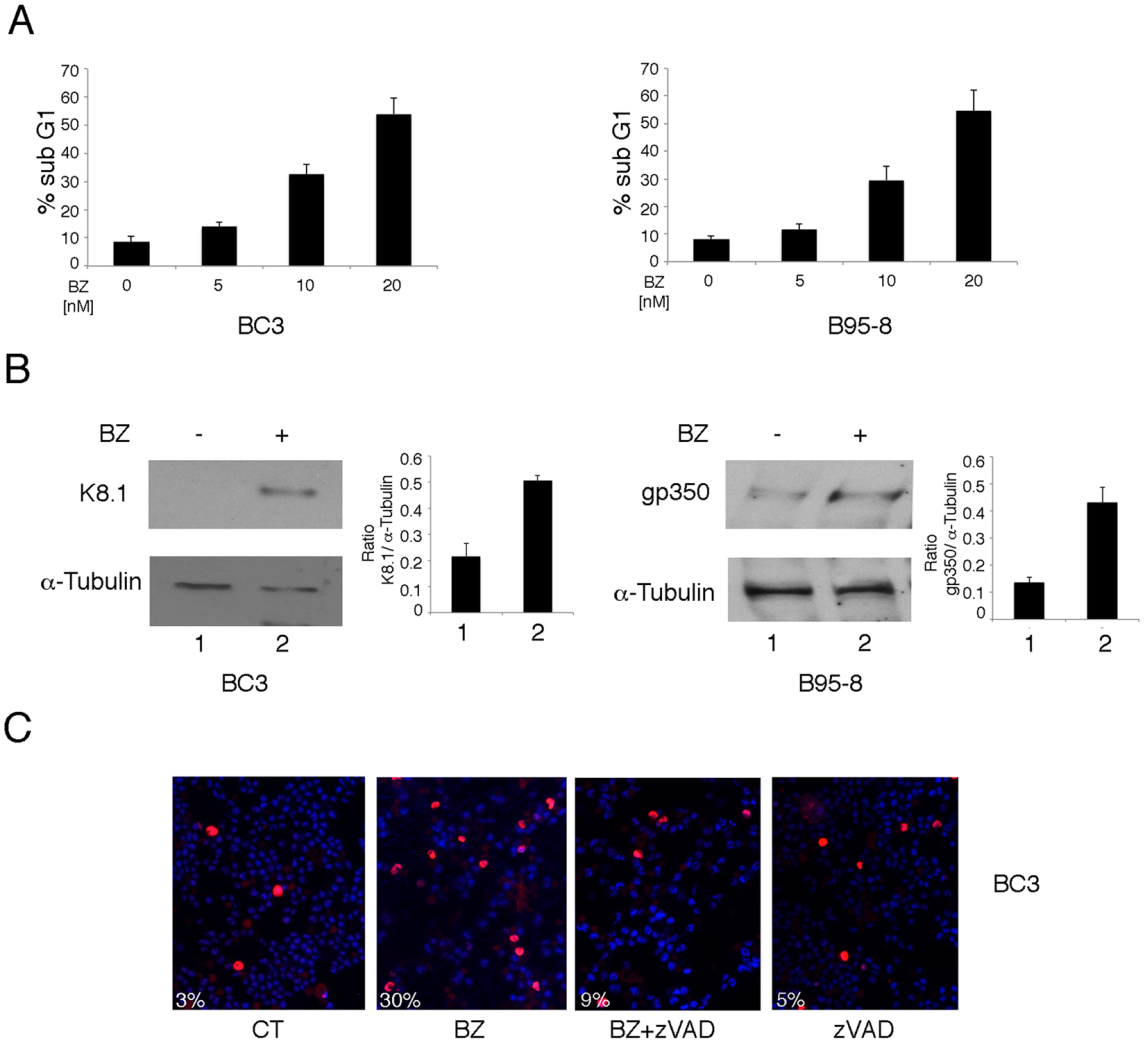
Viral replication is induced by bortezomib in cells undergoing apoptosis. (**A**) BC3 and B95-8 cells were treated with the indicated doses of bortezomib for 24 hours and apoptosis occurrence was evaluated by FACS analysis as sub-G1 percentage. (**B**) BC3 and B95-8 cells undergoing 24 hours of bortezomib treatment were analysed by western blot for the expression of the late viral proteins K8.1 and gp 350 for KSHV and EBV, respectively. α-Tubulin was used as loading control. The histograms represent the mean plus standard deviation (SD) of the densitometric analysis of the ratio of specific proteins on α-Tubulin of three different experiments. (**C**) BC3 cells pre-treated or not with z-VAD (50 μM) were exposed to bortezomib at 20 nM for 24 hours and then analysed for K-bZIP expression by IFA. The percentage of K-bZIP expressing cells is also indicated. DAPI was used as nuclear staining.

PEL cell lines harboring KSHV (BC3 and BCBL1) or lymphoma cells infected by EBV (Raji) or B cells *in vitro* transformed by the virus (B95-8) were treated with bortezomib for the indicated times. We found that in all cell lines, bortezomib induced time-dependent KSHV and/or EBV lytic antigen expression that occurred concomitantly with autophagy activation, as indicated by the viral lytic protein expression that increased at the same times in which microtubule-associated protein 1 A/1B-light chain 3-II (LC3-II) accumulated (Fig. 1A). As control, we performed the same kinetic experiment in mock-treated cell lines and, as expected, neither viral lytic antigens nor LC3-II expression increased over time in the control treated cells (Fig. 1B). Next, a dose-response experiment indicated that viral KSHV and EBV lytic antigen and LC3-II expression were up-regulated as bortezomib dose increased (Fig. 1C). Interestingly, the apoptotic process was induced by bortezomib at the same doses at which it triggered viral replication (Fig. 2A). We also found that the bortezomib-mediated gammaherpesvirus lytic cycle was complete, as both BC3 and B95-8 cells express the KSHV and EBV late lytic antigens K8.1 and gp350, respectively (Fig. 2B). Finally, according to a previous study^25^, when BC3 cells were pretreated with z-VAD, the lytic cycle antigen expression was reduced (Fig. 2C), suggesting that caspase activation play a role in the induction of viral replication.

Altogether, these experiments indicate that gammaherpesvirus lytic antigens and autophagy activation occurred concomitantly, in time- and dose-dependent fashion, in cells harboring latent KSHV or EBV infection undergoing apoptosis upon bortezomib-treatment.

### Autophagy is involved in gammaherpesvirus lytic cycle activation by bortezomib

**Figure 3:**
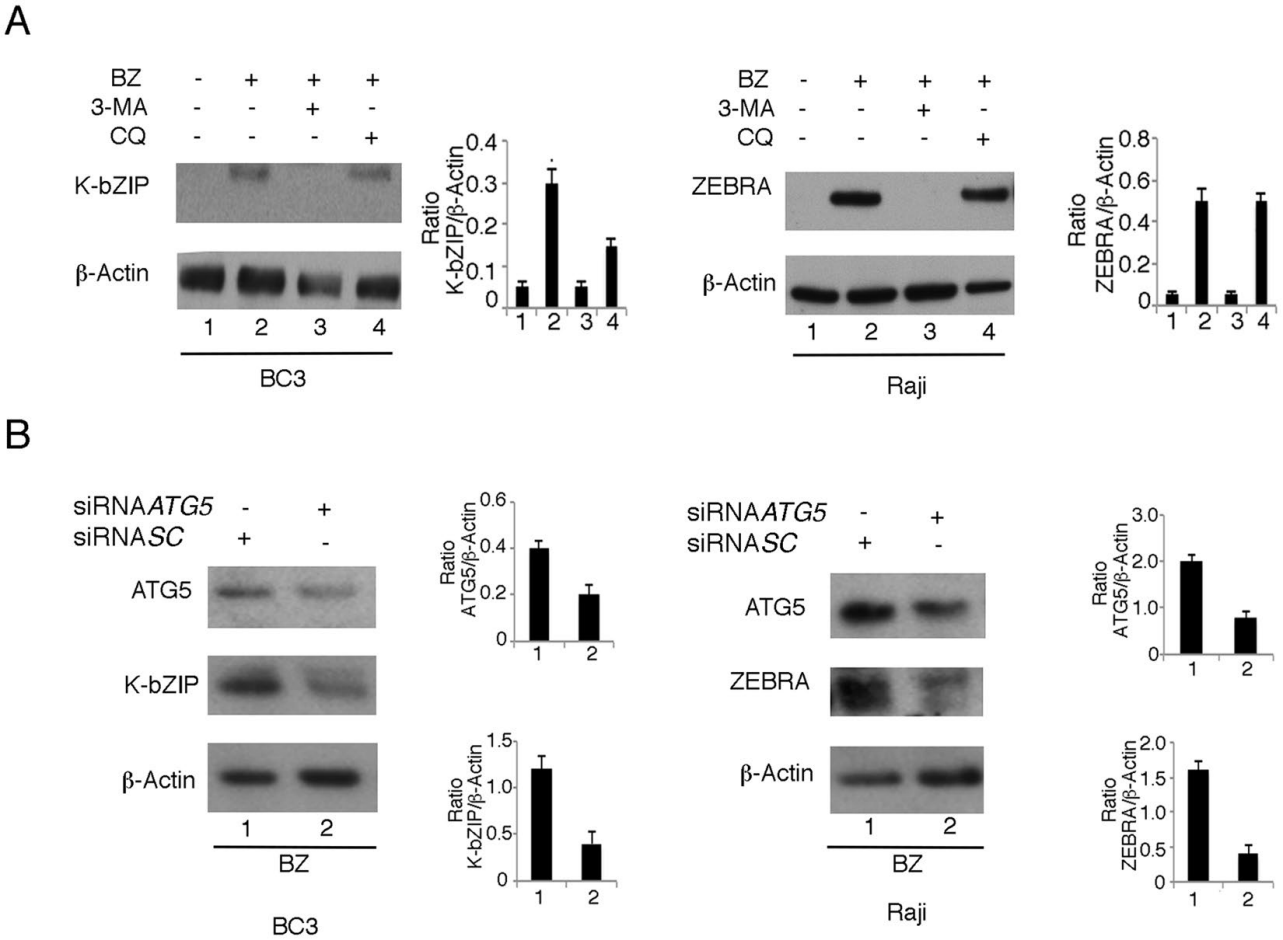
Bortezomib-induced autophagy promotes KSHV and EBV lytic cycle. (**A**) BC3 and Raji cells were treated with bortezomib in the presence or in the absence of 3-Methyladenine (3-MA) (5 mM) or chloroquine^33^ (10 μM) and viral lytic antigen expression (K-bZIP and ZEBRA) was assessed after 24 hours of treatment by western blot analysis. (**B**) BC3 and Raji cells were silenced for ATG5 (siRNA*ATG5*) or scramble-treated (siRNA*SC*) for 48 hours and K-bZIP and ZEBRA expression was analyzed following 24 hours of bortezomib treatment. β-Actin was used as loading control. The histograms represent the mean plus standard deviation (SD) of the densitometric analysis of the ratio of specific proteins on β-Actin of three different experiments.

We then assessed whether autophagy would play a role in bortezomib-induced lytic cycle. As previously reported for TPA/Butyrate (T/B)-induced lytic cycle^15,16^, we found that the inhibition of the first autophagic steps by 3-Methyladenine (3-MA) reduced viral lytic antigen expression induced by bortezomib in BC3 and Raji cells (Fig. 3A). Differently, when autophagy was blocked at the final phases with the lysomonotropic agent chloroquine, that inhibits lysosome acidification, the viral lytic protein expression was slightly affected (Fig. 3A). This suggests that the last autophagic steps do not play a role in the KSHV and EBV replicative process, likely due to the previous finding that autophagy is blocked at the final steps by gammaherpesvirus lytic antigens^15,16^. Next, to exclude off-target effects of pharmacological treatments, we silenced ATG5 by specific siRNA in BC3 and Raji cells before bortezomib treatment. As shown in Fig. 3B, K-bZIP and ZEBRA expression was reduced by ATG5 knock-down in comparison to scrumble siRNA, confirming the importance of the first phases of autophagy in bortezomib-induced lytic cycle.

### Autophagy and gammaherpesvirus lytic cycle induction by bortezomib is dependent on JNK activation in the course of ER stress

**Figure 4:**
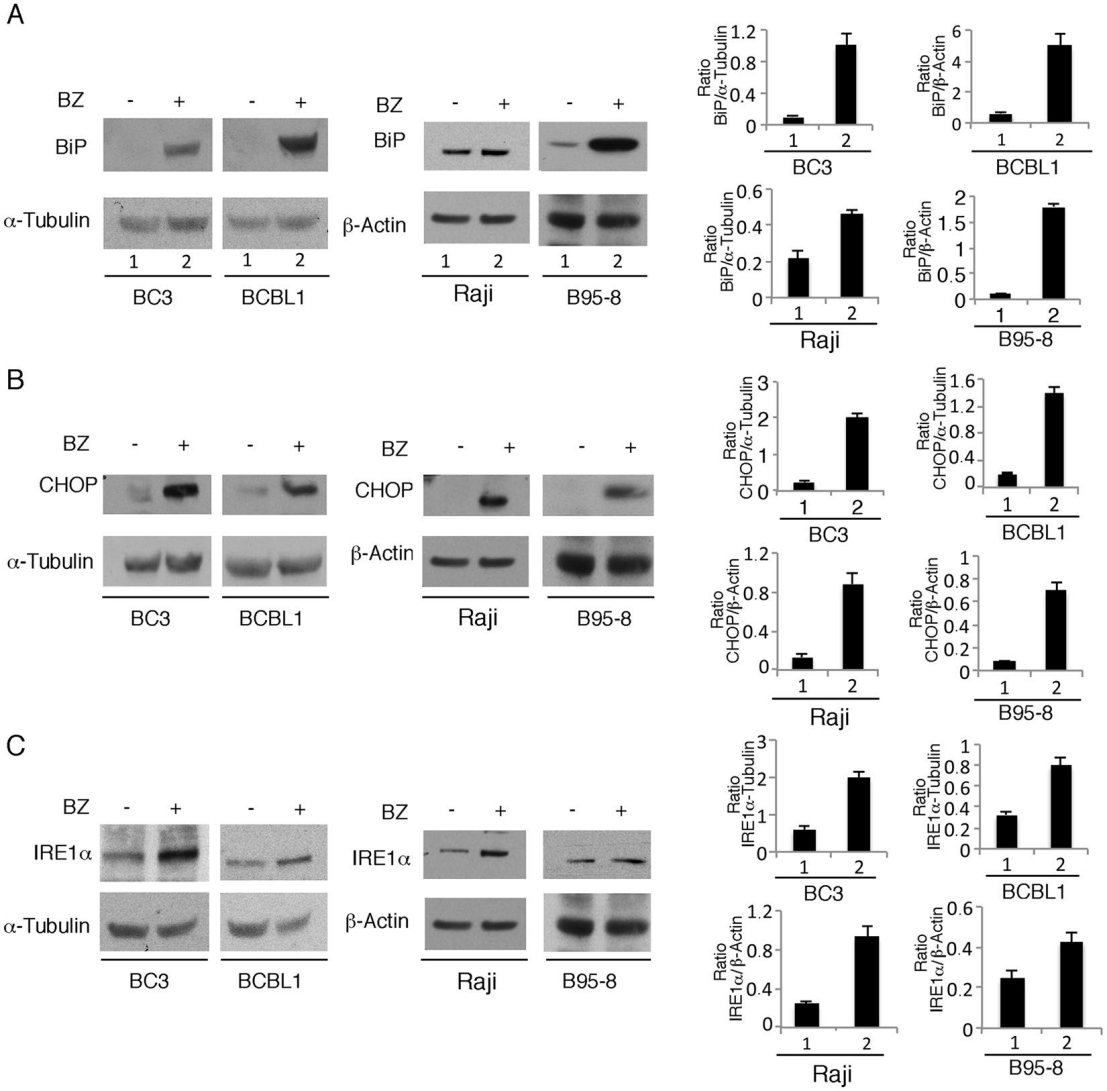
Bortezomib induces ER stress in lymphoma cells in which KSHV or EBV lytic cycle is activated. BC3, BCBL1, Raji and B95-8 cells were treated with bortezomib (BZ) (20 nM) and (**A**) BiP, (**B**) CHOP and **(C)** IRE1α expression was evaluated by western blot after 24 hours. The histograms represent the mean plus SD of the densitometric analysis of the ratio of specific proteins on β-Actin or α-Tubulin of three different experiments.

**Figure 5:**
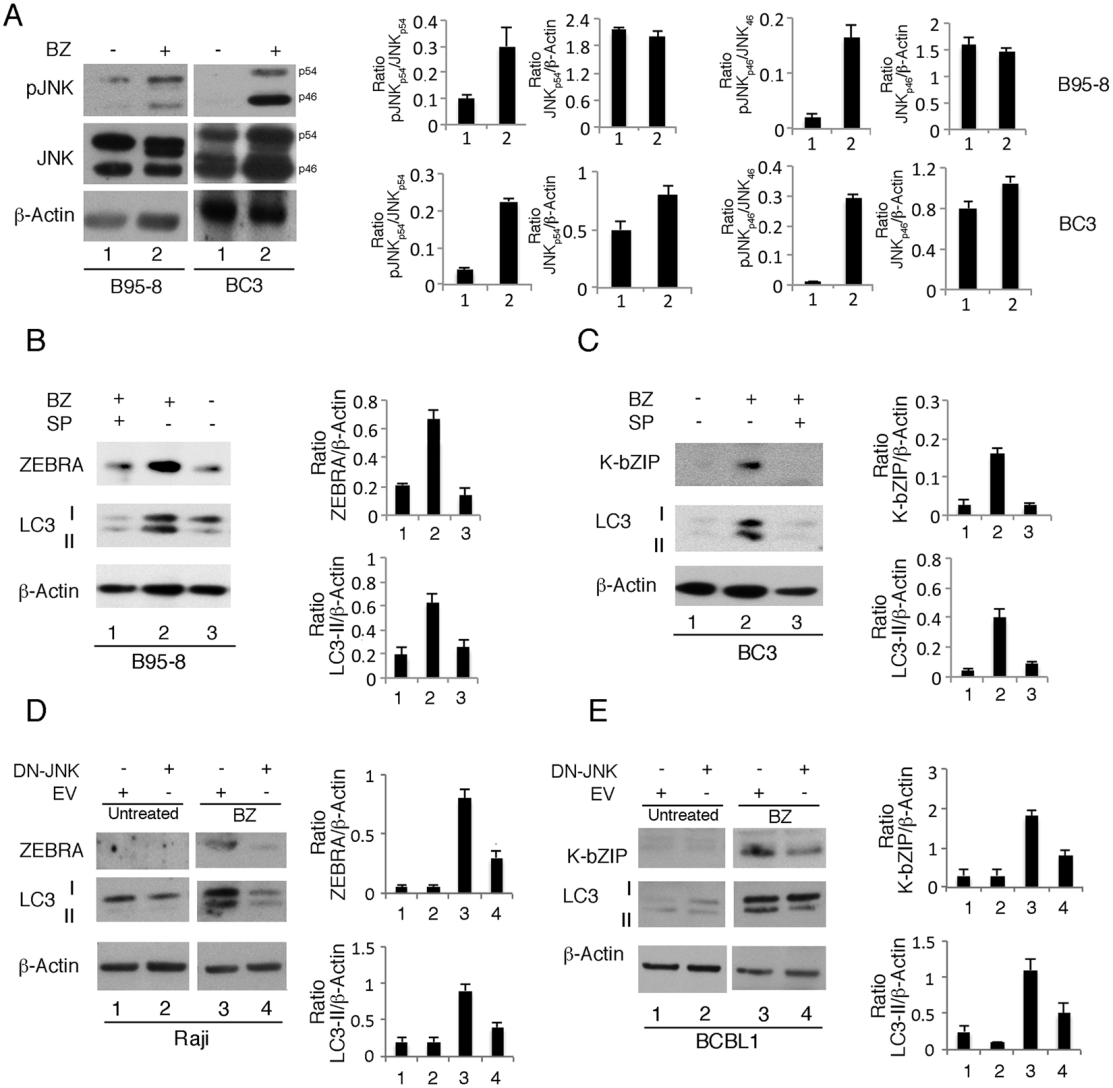
JNK activated by bortezomib promotes gamma-herpesvirus lytic cycle through autophagy induction. (**A**) B95-8 and BC3 cells were treated with bortezomib (BZ) (20 nM) for 24 h and JNK phosphorylation (pJNK, p46 or p54) and total JNK expression (p46 or p54 isoforms) was investigated by western blotting. (**B**) B95-8 and (**C**) BC3 were pre-treated with SP600125 at 20 μM or mock-treated and then, cultured in the presence of bortezomib (BZ) (20 nM) for 24 hrs. EBV or KSHV lytic antigens ZEBRA and K-bZIP as well as LC3-I/II expression was analysed by western blotting. (**D**) Raji and (**E**) BCBL1 cells were transfected with nonphosphorylatable mutant JNK plasmid (DN-JNK) or with empty vector and then treated with bortezomib (BZ) (20 nM) for 24 h. The expression of ZEBRA, K-bZIP and LC3-I/II was evaluated by western blot. The histograms represent the mean plus SD of the densitometric analysis of the ratio of phosphorylated/total proteins or of specific proteins on β-Actin of three different experiments.

Bortezomib has been reported to induce ER stress due to proteasome inhibition and unfolded protein accumulation in the ER^26^. Accordingly, we previously demonstrated that ER stress was induced in a time-dependent manner in PEL cells undergoing bortezomib treatment. In this study, we investigated whether ER stress would occur concomitantly with viral replication and autophagy induction both in KSHV and as EBV-positive lymphoma cells treated with bortezomib^13^. As shown in Fig. 4A, B and C, the expression of BiP, CHOP and IRE1α (indicative of ER stress) increased in all cell lines studied, upon 24 hours of bortezomib treatment. ER stress can activate JNK that may induce autophagy^12,13^. Thus, we next evaluated JNK activation in KSHV and EBV-positive cells treated with bortezomib and its involvement in autophagy induction. As shown in Fig. 5A, although at different extent, JNK was hyper-phosphorylated in BC3 and B95-8 cell lines treated with bortezomib for 24 hours. Next, to investigate the role of JNK in bortezomib-induced KSHV and EBV lytic cycle and autophagy, JNK was inhibited by SP600125 specific inhibitor. We found that it strongly reduced either ZEBRA EBV lytic antigen and K-bZIP KSHV lytic antigen, as well as the autophagic marker LC3-II, in both B95-8 and BC3 cell lines (Fig. 5B and C), suggesting a pivotal role of JNK in promoting both processes. To confirm the importance of JNK in the induction of the viral lytic cycle and autophagy during bortezomib treatment, we transfected Raji and BCBL1 cells with HA-JNK-APF nonphosphorylatable mutant of JNK (DN-JNK) plasmid. As shown in Fig. 5D and E, both viral lytic antigen expression and autophagy was reduced in DN-JNK-transfected lymphoma cells in comparison with empty vector-transfected cells, upon bortezomib treatment. All together these results suggest that JNK promoted autophagy and lytic cycle in bortezomib-treated cells harboring gammaherpesvirus infection.

### JNK is activated in lymphoma cells treated with thapsigargin as well as in stressful conditions in which gamma-herpesvirus reactivation occurs

**Figure 6:**
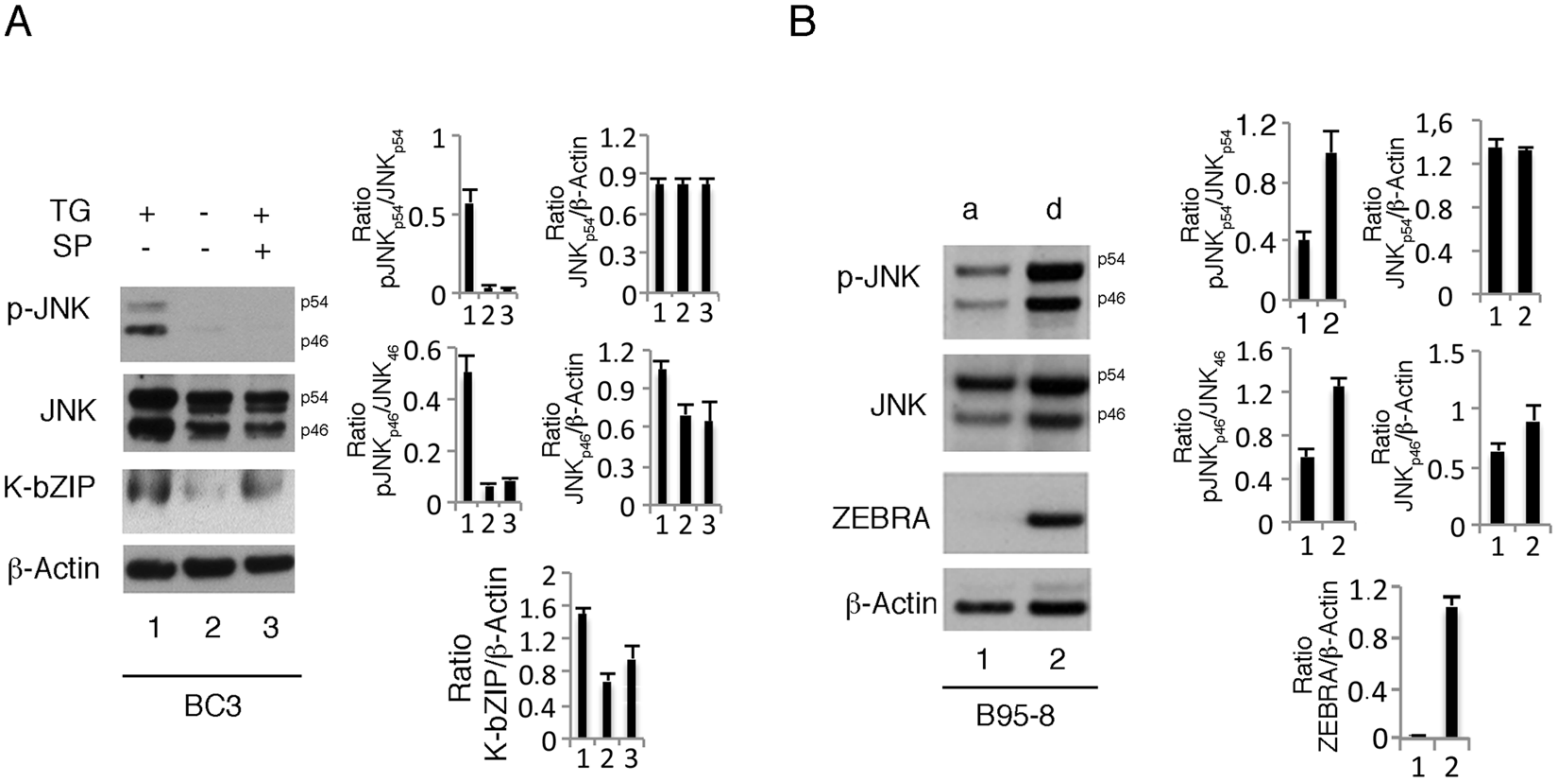
JNK is hyper-phosphorylated in BC3 cells treated with thapsigargin and in B95-8 cells undergoing spontaneous viral replication. (**A**) BC3 cells were treated with thapsigargin (5 μM) for 24 h and JNK phosphorylation, total JNK (p46 or p54) and K-bZIP expression was evaluated by western blot analysis. (**B**) B95-8 adherents (a) or detached (d) were analyzed for JNK phosphorylation, total JNK (p46 and p54 isoforms) and ZEBRA expression by western blotting. The histograms represent the mean plus SD of the densitometric analysis of the ratio of phosphorylated/total proteins or of specific proteins on β-Actin of three different experiments.

We then assessed the activation and the role of JNK in KSHV lytic cycle activation by thapsigargin (Fig. 6A), another ER stressor known to promote gammaherpesvirus reactivation from latency^5,6^. As shown in Fig. 6A, JNK inhibition by SP600125 reduced K-bZIP expression also in this context, suggesting that JNK plays a role also in the activation of viral lytic cycle triggered by this ER stressor. Furthermore, we next investigated whether JNK would be activated in B95–8 cells that detached from the plastic surface (B95-8_d_) and displayed an increased rate of spontaneous viral replication. The results shown in Fig. 6B demonstrated that JNK was hyper-phosphorylated in this cellular fraction (B95-8_d_) showing higher ZEBRA expression, likely because underwent to increased cell stress. All together these results suggest that the activation of JNK could be a common event in B cells undergoing gammaherpesvirus replication, either in spontaneous or induced conditions of cellular stress.

## Discussion

This study shows that the activation of JNK may represent a molecular link between ER stress, autophagy and EBV and KSHV lytic cycle induction by bortezomib. We found indeed that JNK activation promoted both autophagy and viral lytic cycle in cells infected with gammaherpesviruses treated with bortezomib. It has been recently shown that bortezomib in combination with romidepsin induces an autophagic cell death in gastric carcinoma cells by activating MAPKs including JNK^27^. Interestingly bortezomib, already introduced in clinic use against multiple myeloma^28^, seems to be promising also for the therapy of PEL, an aggressive malignancy against which the traditional chemotherapies usually fail. Indeed, it has been reported that this drug can efficiently trigger apoptosis in PEL cells^29^ that we then showed to be an immunogenic apoptosis, leading to the exposure of several damage-associated molecular patterns (DAMPs)^30^. Later on, we showed the bortezomib induced ER stress and a pro-survival autophagy in PEL cells through the activation of JNK^13^. It is important, for cancers harboring onco-viruses such as KSHV and EBV, to investigate whether and how chemotherapies may affect viral replication. Indeed, if in one hand the onset of replication renders more efficient the use of antiviral agents, on the other hand, the replicative process facilitates viral spread, which might support maintenance/progression of gammaherpesvirus-associated cancers^23,24^. Therefore, understanding the molecular mechanisms that regulate viral reactivation in the course of chemotherapy could help to avoid this unwanted effect. In this study, we found that the inhibition of JNK activation and autophagy could restrain lytic cycle induction by bortezomib in both EBV and KSHV-infected B cells. Similarly, we previously observed that the lytic cycle induction by TPA in combination with sodium butyrate (T/B) could be reduced by autophagy inhibition^15,16^. Here, we found that the inhibition of both autophagy and JNK activation reduced KSHV and EBV replication in bortezomib-treated cells. Interestingly, the inhibition of JNK and autophagy was previously shown to increase bortezomib-mediated cytotoxicity against PEL^13^. The finding of this study, showing that such inhibition can also reduce the viral replicative cycle in KSHV as well as in EBV-associated lymphoma cells renders the use of these inhibitors in combination with bortezomib even more promising in anticancer therapy. Gammaherpesvirus replication can be triggered by apoptotic stimuli although apoptosis has been shown to be neither necessary nor sufficient to induce such effect^3^. This study suggests that the activation of JNK and autophagy could represent possible links needed for initiating the viral replicative process in the course of treatment with cytotoxic drugs such as bortezomib. However, according to a previous study^25^, we found that the pan-caspase inhibitor zVAD was able to reduce viral lytic antigen expression. Since gammaherpesvirus replication is lytic, it is quite reasonable that it occurs in cells already committed to die. Indeed, it is possible that viruses exit from dying cells to infect new viable cells from which their survival is totally dependent. Another interesting finding of this study is that gammaherpesvirus replication is regulated by JNK activation also in other than bortezomib-induced stressful conditions. Indeed, we found that JNK was activated also during thapsigargin-induced ER stress and KSHV lytic cycle activation as well as in B95-8 cells undergoing spontaneous *in vitro* EBV replication. Cellular stress represent a physiological stimulus that triggers viral reactivation from latency *in vivo*, thus JNK activation is likely to be involved also in spontaneous gammaherpesvirus *in vivo* replication. Finally, in this study, we showed that although autophagy was involved in gammaherpesvirus replication, the last autophagic steps did not play a role in this process, likely because autophagy is blocked at its final steps during gammaherpesvirus replication^15,24^. It would be interesting to investigate whether JNK or other MAPKs manipulation could be overcame the autophagic block and push the autophagosomes, transporting the viral particles, into the lysosomes for viral degradation. In conclusion, by showing that JNK activation by bortezomib promotes autophagy and KSHV and EBV viral lytic cycle, this study suggests that combining JNK or autophagy inhibitors with bortezomib might be useful to reduce viral spread and likely to improve the outcome of bortezomib therapy against gamma-herpesvirus-associated malignancies.

## Materials and Methods

### Cells

BC3, BCBL1 (human B-cell lines derived from PEL carrying latent KSHV), Raji (human B cell line derived from BL carrying latent EBV) and B95-8 (marmoset B-cell line EBV-infected)^31^ were cultured in RPMI 1640 (Sigma Aldrich), 10% Fetal Bovine Serum (FBS) (Sigma Aldrich), L-glutamine (Aurogene) and streptomycin (100 μg/ml) and penicillin (100 U/ml) (Aurogene) in 5% CO_2_ at 37 °C.

### Cell treatments

BC3, BCBL1, Raji and B95-8 cell lines were treated with bortezomib (BZ) (Santa Cruz Biotechnology Inc.) at 20 nM for 0, 4, 6, 24 hrs or mock treated. A dose-response experiment with 0-10-20 nM (for 24 hours) was also performed in the same cell lines. In some experiments, BC3 were treated with Thapsigargin (5 μM) for 24 hrs. To evaluate the role of autophagy in viral replication, BC3 and Raji cell lines were cultured with bortezomib (20 μM) (Santa Cruz Biotechnology Inc.) in the presence or in the absence of chloroquine (10 μM) (Sigma Aldrich) or with 3-Methyladenine (3-MA) (5 mM) (Santa Cruz Biotechnology Inc.) for 24 hrs^32,33^. To further investigate autophagy, siRNA*ATG5* experiments were performed on the same cell line, as previously reported^15^.

In order to investigate the role of JNK, BC3 and B95-8 cell lines were pre-treated with SP600125 (SP, JNK inhibitor) (Santa Cruz Biotechnology Inc.) at 20 μM or Raji and BCBL1 cells were transfected with HA-JNK-APF nonphosphorylatable mutant of JNK (DN-JNK) plasmid or control vector^13^, and then cultured in presence of bortezomib (BZ) (20 nM) for 24 h.

In some experiments, cells were pretreated for 30 min with z-VAD pan caspase inhibitor (50 μM) (Santa Cruz Biotechnology Inc.) before exposure to bortezomib at 20 nM for 24 hours.

### Antibodies

In this work to evaluate the expression of viral proteins we used: mouse monoclonal anti-K-bZIP (1:500) (Santa Cruz Biotechnology Inc.), rabbit polyclonal anti-ORF50-RTA (1:500) (kindly provided by Prof. D. Ganem)^34^, mouse monoclonal anti-ZEBRA (1:100) (Santa Cruz Biotechnology Inc.), anti-K8.1 (1:400) (Advanced Biotechnologies, 13-213-100) and anti gp350 (1:500) (ATCC 72A1). We also used the following primary antibodies: rabbit polyclonal anti-BiP (1:100) (Cell Signaling), mouse monoclonal anti-CHOP (1:100) (Cell Signaling), mouse monoclonal anti-CHOP (1:100) (Santa Cruz Biotechnology Inc.), mouse monoclonal anti-IRE1α (1:100) (Santa Cruz Biotechnology Inc.), rabbit polyclonal anti-JNK (p46 and p54) (1:500) (Cell Signaling), rabbit polyclonal anti-pJNK (p46 and p54) (1:300) (Cell Signaling).

To study autophagy we used a rabbit polyclonal anti-LC3 (1:1000) (Novus Biologicals) and a rabbit polyclonal anti-ATG5 (1:500) (Novus Biologicals). Monoclonal mouse anti-β-actin (1:10000) (Santa Cruz Biotechnology Inc.) and monoclonal mouse anti-α-Tubulin (1:10000) (Santa Cruz Biotechnology Inc.) antibodies were used as a marker of equal loading. Goat polyclonal anti-mouse IgG-HRP (Santa Cruz Biotechnology Inc) and anti-rabbit IgG-HRP (Santa Cruz Biotechnology Inc.) were used as secondary antibodies. All the primary and secondary antibodies used in this study were diluted in a PBS- 0.1% Tween 20 solution containing 3% BSA.

### Western blot

Cells (1 × 10^6^) were washed twice with 1X PBS solution and centrifuged at 1500 rpm for 5 minutes. The pellet was lysed in a RIPA buffer containing 150 mM NaCl, 1% NP-40, 50 mM Tris-HCl (pH 8), 0.5% deoxycholic acid, 0.1% SDS, protease and phosphatase inhibitors. Then, 20 µg of protein lysates were subjected to electrophoresis on 4–12% NuPage Bis-Tris gels (Life Technologies) according to the manufacturer’s instruction. Then, the gels were transferred to Nitrocellulose Membranes (Biorad) for 2 hrs in Tris-Glycine. The membranes were blocked in PBS-0.1% Tween20 solution containing 3% of BSA, probed with specific antibodies and developed using ECL Blotting Substrate (Advansta)^35^.

### ATG5 Knockdown by small interfering RNA

The knockdown of ATG5 (Santa Cruz Biotechnology, sc-41445) was performed in BC3 and Raji cell lines using specific small interfering RNA duplex. The day before transfection, 3 × 10^5^ cells were seeded in 12-well culture plates in RPMI medium without antibiotics. Subsequently, 75 pmol of siRNA duplex and 7.5 µl of Lipofectamine 2000 Transfection Reagent (Life Technologies, 11668027) were diluted in Optimem medium (Life Technologies, 31985062) and added to the cells for 48 hrs. Then, cells were treated with bortezomib (BZ) at 20 nM for 24 hrs.

The transfection efficiency was evaluated by a Fluorescein Conjugate-A siRNA (Santa Cruz Biotechnology, sc-36869), which was also used as a scrambled control.

### Transfection and plasmids

We performed a transient transfection using HA-JNK-APF nonphosphorylatable mutant of JNK (DN-JNK) plasmid or control vector in Raji and BCBL1 cell lines. Briefly, 5 × 10^5^ cells were seeded into 24-well plates in RPMI medium without antibiotics and the day after, cells were transfected using 1 µg of plasmid DNA/well and with 2.5 µl of Lipofectamine 2000 transfection reagent 2000/well for 24 hrs, according to the manufacture’s instructions. Subsequently, cells were treated with bortezomib (BZ) (20 nM) for 24 hours and a western blot analysis was performed to evaluate ZEBRA (Raji cells) or K-bZIP (BCBL1 cells) and LC3-II proteins expression.

### Densitometric Analysis

The quantification of proteins bands was performed by densitometric analysis using the Image J software (http://imagej.nih.gov).

### Statistical analysis

Results are represented by the mean ± standard deviation (SD) of at least three independent experiments.
